# Survival Outcomes and Patterns of Recurrence in Patients with Stage III or IV Oropharyngeal Cancer Treated with Primary Surgery or Radiotherapy

**DOI:** 10.7759/cureus.713

**Published:** 2016-07-26

**Authors:** Brock J Debenham, Robyn Banerjee, Heather Warkentin, Sunita Ghosh, Rufus Scrimger, Naresh Jha, Matthew Parliament

**Affiliations:** 1 Department of Oncology, University of Alberta; 2 Department of Oncology, University of Calgary

**Keywords:** oropharyngeal, hpv, oropharyngeal cancer, surgery, radiation, radiotherapy, recurrence, patterns of failure, failure

## Abstract

**Purpose:**

To compare and contrast the patterns of failure in patients with locally advanced squamous cell oropharyngeal cancers undergoing curative-intent treatment with primary surgery or radiotherapy +/- chemotherapy.

**Methods and materials:**

Two hundred and thirty-three patients with stage III or IV oropharyngeal squamous cell carcinoma who underwent curative-intent treatment from 2006-2012, were reviewed. The median length of follow-up for patients still alive at the time of analysis was 4.4 years. Data was collected retrospectively from a chart review.

**Results:**

One hundred and thirty-nine patients underwent primary surgery +/- adjuvant therapy, and 94 patients underwent primary radiotherapy +/- chemotherapy (CRT). Demographics were similar between the two groups, except primary radiotherapy patients had a higher age-adjusted Charleston co-morbidity score (CCI). Twenty-nine patients from the surgery group recurred; 15 failed distantly only, seven failed locoregionally, and seven failed both distantly and locoregionally. Twelve patients recurred who underwent chemoradiotherapy; ten distantly alone, and two locoregionally. One patient who underwent radiotherapy (RT) alone failed distantly. Two and five-year recurrence-free survival rates for patients undergoing primary RT were 86.6% and 84.9% respectively. Two and five-year recurrence-free survival rates for primary surgery was 80.9% and 76.3% respectively (p=0.21). There was no significant difference in either treatment when they were stratified by p16 status or smoking status.

**Conclusions:**

Our analysis does not show any difference in outcomes for patients treated with primary surgery or radiotherapy. Although the primary pattern of failure in both groups was distant metastatic disease, some local failures may be preventable with careful delineation of target volumes, especially near the base of skull region.

## Introduction

Locally advanced oropharyngeal cancers are increasing in incidence. Although most centers throughout Canada and the United States of America (USA) favor treating these malignancies with an organ-preservation approach using combined chemoradiotherapy (CRT) [1], some centers, including ours, have a large experience treating with primary surgery followed by adjuvant therapy [2]. Recently our center reported outcomes of our experience from the years 1998 to 2009, which appeared to show an improved disease-free survival at two years for surgery as a primary treatment compared to CRT (73.7% vs. 57.4%) [2]. Previous studies from Stanford and others have consistently reported 3-4 year local control rates for patients treated with CRT of 90% or higher, and 3-year disease-free survival rates of approximately 80% [3-8].

Due to the large discrepancy in our outcomes compared to other large academic centers, we undertook a quality assurance study looking at stage-matched patients with locally advanced oropharyngeal cancer undergoing either primary surgery or radiotherapy with an emphasis on disease-free survival, overall survival, and patterns of recurrence.

## Materials and methods

Ethics approval was obtained before initiating this study through the Health Research Ethics Board of Alberta – Cancer Committee (ETH#26196). The patient list was obtained from the Alberta Cancer Registry (ACR). The list was created by searching for all stage III and IV squamous cell cancers (SCC) of the oropharynx treated with primary surgery +/- adjuvant therapy or radiotherapy +/- chemotherapy. The timelines used were from January 1, 2006 to December 31, 2012 and the location was Northern Alberta. All patients had CT or PET imaging of the neck and chest prior to initiation of curative-intent therapy, as well as a formal quadroscopy for biopsy of the primary site of disease.

An initial list of 333 patients was obtained from the ACR. A comprehensive chart review was undertaken, and a database was populated. A final list of 233 patients who underwent non-clinical trial, curative-intent treatment were included in the analysis. The median length of follow-up for patients still alive at the time of analysis was 4.4 years. Reasons for exclusion of the other 100 patients from the ACR were as follows: 27 patients had a non-oropharyngeal primary tumor; 27 had palliative-intent treatment (radiotherapy, chemotherapy, or best supportive care); 22 had metastases at diagnosis; 15 had their primary treatment outside of Northern Alberta; six had recurrent disease from a previous head and neck cancer (prior to 2006); two of them had Stage I or II disease; one had synchronous head and neck (H&N) primaries; one had a non-SCC cancer; and one had been included in the registry twice.

### Statistical analysis

Patient demographics, treatment factors, follow-up dates, imaging results, and pathology results were collected and anonymized. Summary statistics were calculated, including mean and standard deviations for continuous variables, and frequency and percentages for categorical variables. Recurrence-free survival (RFS) and overall survival (OS) was measured from the date of diagnosis to the date of recurrence or death. Kaplan–Meier estimates of the median RFS & OS and 95% confidence interval (95% CI) were obtained. Logistic regression was used to explore the association between factors commonly available at the time of consultation (age, histology, PS, gender) as well treatment factors for both surgery and radiation. After univariate analysis, variables significant at the p < 0.10 level were entered into multivariate models. Final models selected variables significant at the p < 0.05 level. All analyses were conducted using SAS version 9.3, with p < 0.05 indicating statistical significance.

## Results

### Patient demographics 

Patient demographics were analyzed and summarized in Table 1 below.

**Table 1:** Patient Demographics

	Primary Surgery (n=139)	Primary Radiation (n=94)	p-value
Male/Female	121/18	82/12	p=0.88
Age-adjusted Charleston Co-morbidity Index (median)	3 (95% CI 3-4)	4 (95% CI 3-4)	p=0.046
Age at Diagnosis (median)	56 (95% CI 54-57)	56 (95% CI 54-59)	p=0.19
AJCC Stage	III – 21 (15.1%) IVA – 104 (74.8%) IVB – 14 (10.1%)	III – 14 (14.9%) IVA – 62 (65.9%) IVB – 18 (19.1%)	p=0.13
Clinical T-Stage (RT) Pathologic T-Stage (Surgery)	T1 (21.6%) T2 (35.3%) T3 (20.2%) T4a (21.6%) T4b (1.4%)	T1 (40.4%) T2 (21.3%) T3 (20.2%) T4a (11.7%) T4b (6.4%)	p=0.06
Clinical N-stage (RT) Pathologic N-Stage (Surgery)	N0 (6.5%) N1 (13.8%) N2a (11.6%) N2b (33.3%) N2c (28.3%) N3 (6.5%)	N0 (1.1%) N1 (14.9%) N2a (18.1%) N2b (31.9%) N2c (19.1%) ​N3 (14.9%)	p=0.14
Smoking Status	Lifelong non-smoker - 30 (21.6%) Former smoker – 71 (51.1%) Current smoker – 37 (26.6%) Unknown – 1 (0.7%)	Lifelong non-smoker – 19 (20.2%) Former smoker – 41 (43.6%) Current smoker – 34 (36.1%) Unknown – 0 (0%)	p=0.38
P16 Status	Positive – 25 (18.0%) Negative – 8 (5.7%) Unknown – 106 (76.3%)	Positive – 26 (27.7%) Negative – 5 (5.3%) Unknown – 63 (67.0%)	p=0.21
Time from Diagnosis to Initial Treatment (mean, days)	74.6	84.4	p=0.03

### Primary surgery

One hundred thirty-nine patients underwent primary surgery. Seventeen underwent surgery alone, 27 underwent surgery plus adjuvant radiotherapy (SRT), and 95 underwent surgery plus adjuvant chemoradiotherapy (SCRT). The reasons for patients who had surgery alone and did not receive any adjuvant treatment included patient refusal (n=6), patients were not offered adjuvant therapy (n=3), metastases presented after surgery but prior to starting adjuvant therapy (n=4), patient died prior to starting adjuvant therapy (n=3), or poor performance status after surgery (n=1).

Patients at our center are routinely offered concurrent chemotherapy post-surgery for intermediate risk factors such as T3/T4 disease, perineural invasion (PNI), lymphovascular space invasion (LVSI), or node positive disease rather than only in patients with positive margins or extracapsular extension [9, 10]. There was not a significant difference in RFS or OS in patients who received SRT or SCRT. Patients began their adjuvant treatment, on average, 56 days (95% CI 53-59 days) post-surgery, with only 8% of patients starting within our guideline of six weeks post-surgery [11].

Twenty-nine patients from the surgery group recurred; 15 failed distantly only, seven failed locoregionally, and seven failed both distantly and locoregionally.

Regression analysis was performed, and on univariate analysis, the following variables were found to be significant, as listed below in Table 2.

**Table 2:** Univariate analysis for risk factors for recurrence in patients undergoing primary surgery.

Factor	Hazard Ratio	p-value
Nodes Positive (0, <5, >5)	> 5 nodes - 5.08 (95% CI 2.31-11.1)	p<0.0001
Age Adjusted CI		NS
Age		NS
AJCC Stage		NS
Chemotherapy Type (SCRT only)	Carboplatin – 3.35 (95% CI 1.29-8.64)	p=0.013
Chemotherapy Schedule (Weekly vs every 3 weeks) (SCRT only)	Weekly – 4.40 (95% CI 1.57-12.29)	p=0.003
Radiation Dose (<6000, 6000-6600, >6600)		NS
ECE status	4.23 (95% CI 1.99-9.53)	p=0.0002
Gender	Female 2.61 (95% CI 1.12-6.10)	p=0.04
LVI status	2.15 (95% CI 1.03-4.50)	p=0.04
Margin status	4.11 (95% CI 1.92-8.83)	p=0.001
P16	P16 neg 4.11 (95% CI 1.42-11.80)	p=0.02
pN status	N2c 5.53 (95% CI 2.64-11.6)	p<0.0001
pT status	T3 4.09 (95% CI 1.58 – 10.55) T4a 4.68 (95% CI 1.85-11.83) T4b 55.3 (95% CI 5.67-541.61)	p=0.0004
Smoking status		NS
Time from diagnosis to surgery		NS
Time from surgery to start of radiotherapy (> 6 weeks vs < 6 weeks)		NS
Grade 3	3.07 (95% CI 1.40 – 6.73)	p=0.0052
PNI status	2.30 (95% CI 1.20-4.42)	p=0.013

These variables were then entered into a multivariable analysis. For SCRT patients, chemotherapy schedule was not significant in the multivariate model. For all surgery patients combined, the following variables were significant on multivariate analysis, as listed in Table 3.

**Table 3:** Multivariate analysis for risk factors for recurrence in patients undergoing primary surgery

Factor	Hazard Ratio	p-value
Nodes Positive (0, <5, >5)	> 5 nodes - 4.72 (95% CI 1.59-13.96)	p=0.0054
Gender	Female – 5.08 (95% CI 2.03-12.74)	p=0.0005
P16 negative	4.44 (95% CI 1.92-10.24)	p=0.0005
pT4b	46.98 (95% CI 4.04-546.14)	p=0.0001
Chemotherapy (SCRT only)	Carboplatin – 3.35 (95% CI 1.29-8.64)	p=0.013

### Primary radiotherapy

Ninety-four patients underwent CRT (n=84) or RT alone (n=7). Patients who underwent RT alone did so for the following reasons: four refused chemotherapy, two patients were not chemotherapy candidates, and one patient was not offered a chemotherapy consultation. Our standard dose fractionation at our center is to deliver 6600 cGy over 30 daily fractions, based on RTOG 00-22 [12]. Univariate analysis was performed for risk factors for recurrence, and the results are summarized in Table 4.

**Table 4:** Univariate analysis for risk factors for recurrence in patients undergoing primary RT

Factor	Hazard Ratio	p-value
Age Adjusted CCI		NS
Age		NS
AJCC Stage	IVB – 5.72 (95%CI 1.93 – 16.96)	p=0.0017
Chemotherapy Type (CRT only)		NS
Chemotherapy Schedule (Weekly vs every 3 weeks) (CRT only)		NS
Radiation Dose (<6000, 6000-6600, >6600)		NS
Gender		NS
Persistent disease after primary RT treatment	9.14 (95% CI 3.07-27.21)	p=0.0001
P16		NS
cN status	N3 - 5.23 (95% CI 1.76 – 15.45)	p=0.003
cT status		NS
Smoking status		NS
Time from diagnosis to RT		NS
Grade		NS

The significant variables were entered into multivariate analysis. The results are summarized below in Table 5.

**Table 5:** Multivariate analysis for risk factors for recurrence in patients undergoing primary RT

Factor	Hazard Ratio	p-value
Stage	IVB – 4.85 (95%CI 1.61 – 14.58)	p=0.0051
Persistent disease after RT	7.70 (95% CI 2.55-23.22)	p=0.0003

### Patterns of recurrence

Twelve patients recurred who underwent chemoradiotherapy; ten distantly alone, and two locoregionally. One patient who underwent RT alone failed distantly. Eighteen patients who recurred underwent surgery followed by chemoradiotherapy: 11 distantly alone, two locoregionally alone, and five locoregionally and distantly. Five patients failed who underwent surgery followed by radiation alone: one distantly, three locoregionally, and one locoregionally and distantly. Six patients failed who underwent surgery alone; three distantly, two locoregionally, and one locoregionally and distantly.

For the patients that received radiotherapy as part of their treatment, and failed locally or locoregionally, we analyzed their radiotherapy plans to look at the location of recurrence versus the dose in the region. The results are summarized in Table 6.

**Table 6:** Review of locoregional failures radiotherapy plans

Case	Treatment	Stage/risk factors	Failure Location	Notes
1	SCRT	T4aN2b, positive surgical margins	Base of skull/pterygoid plates	Patient terminated RT early, received 50.4Gy/28 to recurrent area
2	SRT	T2N2c	Sphenoid bone	No coverage of base of skull despite level 2 nodes positive
3	SRT	T2N3, positive margins	Near parotid	Recurrence in 60 Gy region (no RT boost or chemo (poor KPS))
4	SRT	T1N2a, positive margins, ECE	High level 2	High level 2 not covered despite positive lymph node in level 2, marginal miss
5	SCRT	T4aN2c, ECE	Neck	Only completed 48 Gy, quit RT
6	CRT	T3N3	Neck	In high dose RT area
7	CRT	T1N3	Neck	In high dose RT area

**Figure 1:**
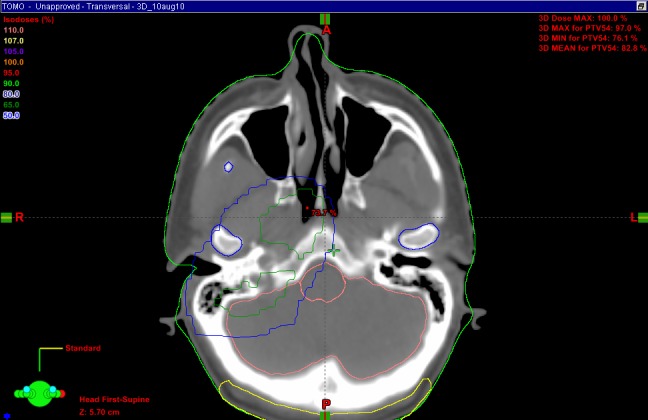
Radiotherapy plan, Case 4, marginal miss, poor coverage of high level 2/base of skull. The plan shows poor coverage (covered by less than the 95% isodose line) at the high level 2 neck lymph nodes.

**Figure 2:**
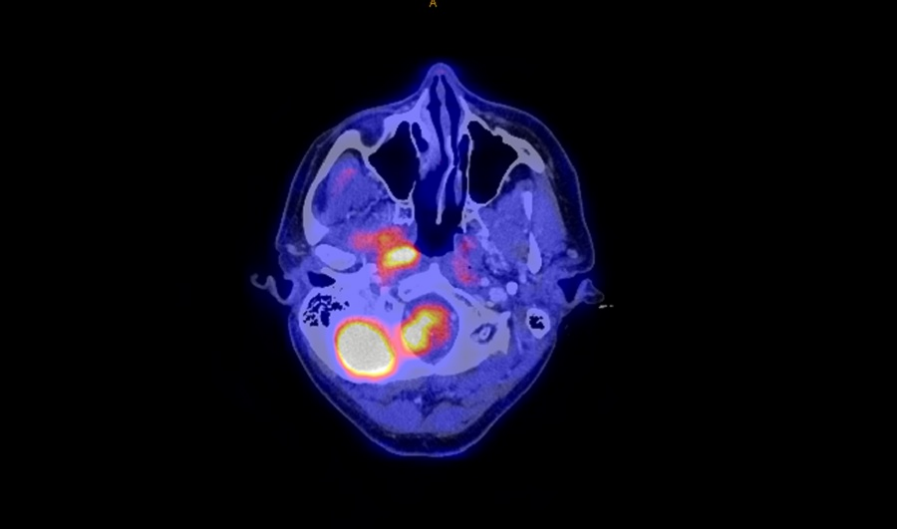
PET scan of recurrence, Case 4, marginal miss, poor coverage of high level 2/base of skull.

As an example, the marginal miss in Case 4 is demonstrated in Figures 1-2 below.

### Recurrence-free survival comparison

**Figure 3:**
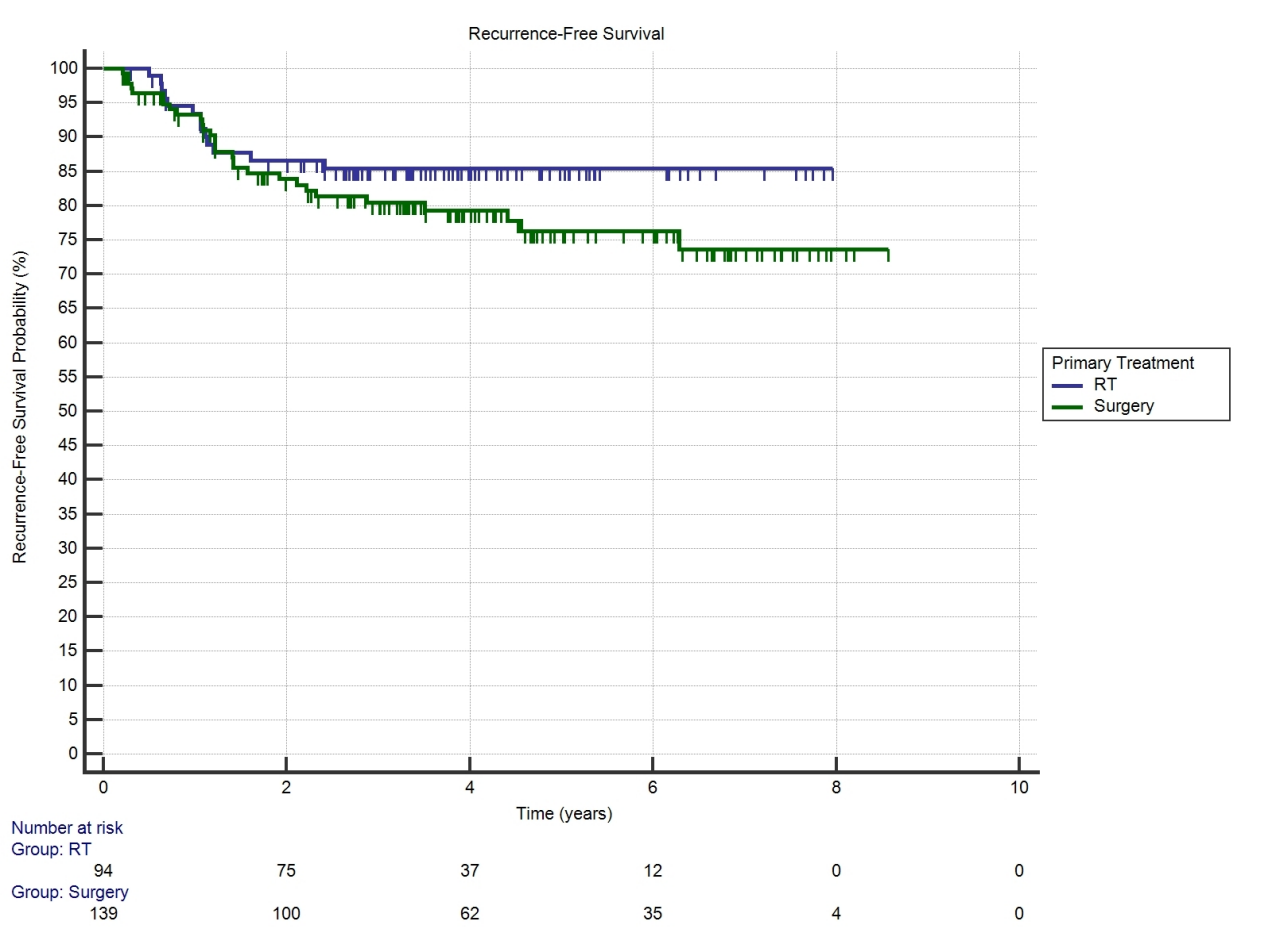
Kaplan-Meier Recurrence-Free Survival for Primary Surgery vs Primary RT

Two & five-year recurrence-free survival rates for patients undergoing primary RT was and 86.6% and 84.9% respectively. Two and five-year recurrence-free survival rates for primary surgery was 80.9% and 76.3% respectively. There was no significant difference in either treatment when stratified by p16 status or smoking status. The Kaplan-Meier estimate of recurrence-free survival is shown in Figure 3 (p = 0.21).

### Overall survival comparison

**Figure 4:**
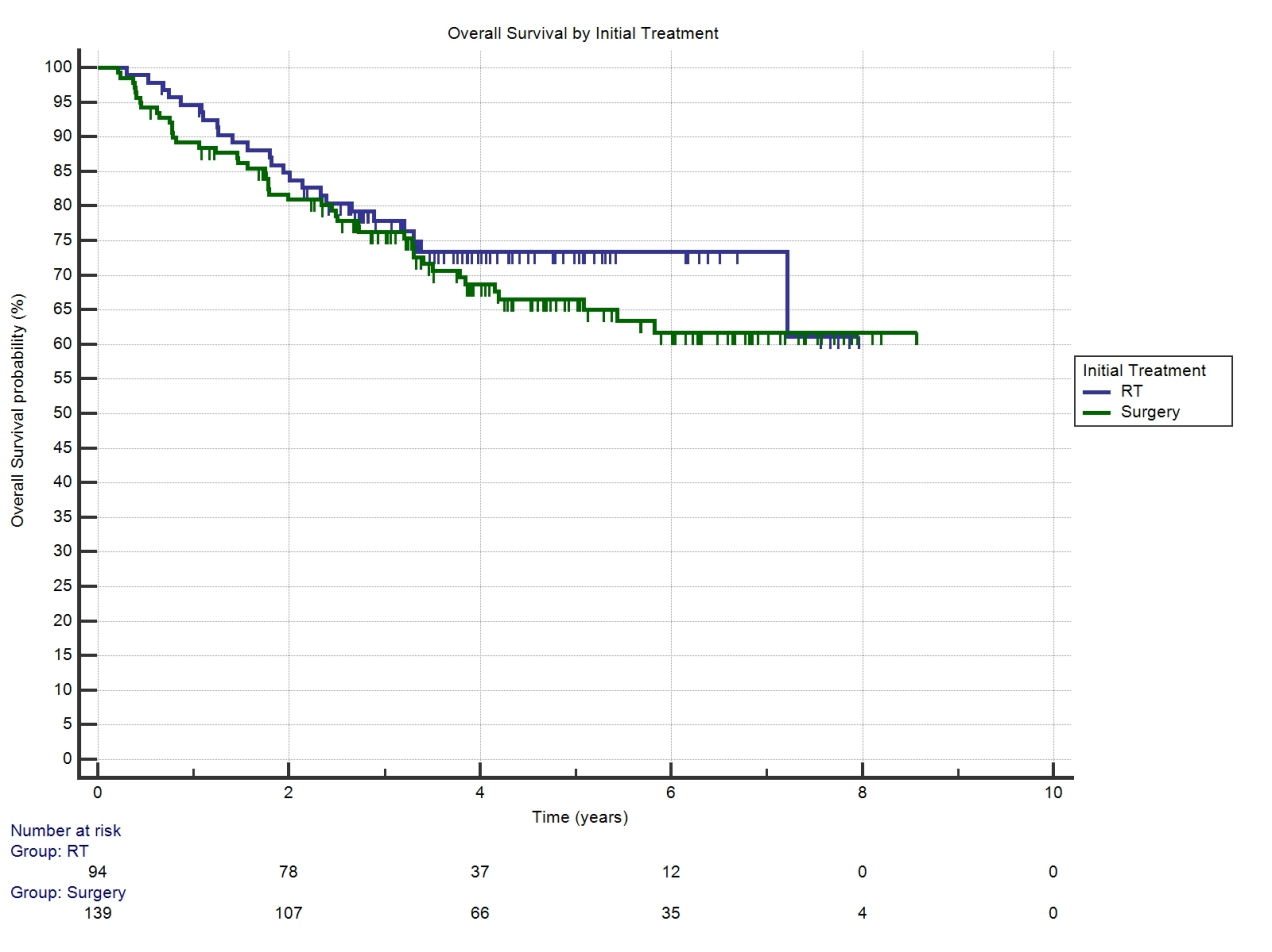
Kaplan-Meier Overall Survival for Primary Surgery vs Primary RT

Two & five-year overall survival rates for primary RT was 86.6% and 73.4% respectively. Two & five-year overall survival rates for primary surgery was 83.9% and 66.5% respectively (p=0.38). There was no significant difference in either treatment when stratified by p16 status or smoking status. The Kaplan-Meier estimate of recurrence-free survival is shown in Figure 4 (p=0.38)

### Causes of death

Twenty-four patients died in the primary RT group (25.5%). Five died of non-cancer causes (20.8%), 11 died of oropharyngeal cancer (45.8%), and eight died of new cancer primaries, the majority being biopsy-confirmed lung cancer (32.0%). Forty-six patients died in the primary-surgery group (33.1%). Sixteen (34.8%) died of non-cancer causes, 24 died of oropharyngeal cancer (52.2%), and six died of a new cancer primary (13.0%).

## Discussion

Our results are consistent with other large academic centers in patients who undergo CRT as primary treatment for locally advanced oropharyngeal carcinoma with two and five-year RFS rates of 86.6% and 84.9%. In comparison to our centre’s previously published results, we found that the percentage of patients undergoing RT alone was not as high (18.3% in previous results vs 3% in this cohort) [2]. This likely reflects the fact that patients receiving RT alone was likely palliative, and these patients should have been removed from the previous study.

Weaknesses of this study include bias in treatments, as patients who underwent primary RT compared to surgery had higher Charleston Co-Morbidity Index (CCI) [13], and a higher proportion of T4b disease. We are missing human papilloma virus (HPV) status on the majority of our patients, as our centre did not routinely test for p16 status until 2010/2011, which limits comparisons on comparing modalities when stratifying by HPV status. Additionally, we have no data in regards to functional outcomes of our patients, or the cost difference in treatment between the two groups.

Although the dominant pattern of failure for patients treated with both primary surgery and radiotherapy remains a distant failure, it may have been possible to prevent some local recurrences with adjustment of the radiotherapy plans. Specifically, we had two recurrences at the base of the skull and one near the parotid gland in primary surgery patients who underwent adjuvant treatment. This phenomenon has been described before by Eisbruch et al. [14], therefore, it is important to ensure that this coverage is achieved during radiotherapy planning and QA processes. There were more local recurrences in the surgery group compared to the radiotherapy groups in our study. We do not have a good explanation for this, except perhaps that after surgery oxygenation to the tumor bed may be altered, and perhaps adjuvant radiotherapy is not as effective with the altered oxygenation to the post-surgical bed.

The primary pattern of failure in both primary surgery or radiotherapy patients was distant. This pattern was in many other studies. Results from RTOG 0234 demonstrated a decreased rate of distant metastatic disease in patients receiving docetaxel chemotherapy rather than standard cisplatin chemotherapy [15]. This hypothesis is being further tested in high-risk postoperative patients in RTOG 1216, which is currently open to accrual [16]. Our standard chemotherapy offered to these patients may change in the future based on the results of RTOG 1216, and will hopefully alter the patterns of failure for these patients.

## Conclusions

Our analysis does not show any difference in outcomes for patients treated with primary surgery or radiotherapy. Although the primary pattern of failure in both groups was distant metastatic disease, some local failures may be preventable with careful delineation of target volumes, especially near the base of skull region.
